# Acute Low-Dose Hydralazine-Induced Lupus Pneumonitis

**DOI:** 10.1155/2017/2650142

**Published:** 2017-08-08

**Authors:** Sarah K. Holman, Donique Parris, Sarah Meyers, Jason Ramirez

**Affiliations:** ^1^Notre Dame of Maryland University School of Pharmacy, 4701 North Charles St, Bunting 103, Baltimore, MD 21210, USA; ^2^University of Maryland School of Medicine, Baltimore, MD 21201, USA; ^3^Department of Family and Community Medicine, University of Maryland Medical Center, Baltimore, MD 21201, USA; ^4^Department of Family and Community Medicine, University of Maryland School of Medicine, Baltimore, MD 21201, USA

## Abstract

A 35-year-old female was started on hydralazine 10 mg orally three times a day for treatment of postpartum hypertension. Three months later, after multiple unsuccessful courses of prednisone and antibiotics for presumed pneumonia and asthma exacerbations, her respiratory symptoms progressed in severity and she developed resting hypoxia. Previous diagnostic work-up included spirometry with a restrictive pattern, chest CT showing bilateral basilar consolidation, negative BAL, and nonspecific findings on lung biopsy of mild inflammatory cells. Review of systems was positive for arthralgia, lymphadenopathy, paresthesia, and fatigue that began four weeks after starting hydralazine. A clinical diagnosis of hydralazine-induced lupus (HIL) with pneumonitis was made. Antihistone antibodies were positive supporting a diagnosis of HIL. Management included cessation of hydralazine and a prolonged steroid taper. Within days, patient began improving symptomatically. Six weeks later, CT chest showed complete resolution of infiltrates. Genetic testing revealed she was heterozygous for N-acetyltransferase 2 (intermediate acetylator). Drug-induced lupus should be considered in patients with lupus-like symptoms taking medications with a known association. While the majority of HIL cases occur with high doses and prolonged treatment, cases of low-dose HIL have been reported in patients who are slow acetylators.

## 1. Introduction

Drug-induced autoimmunity (DIA) refers to an immune mediated disease process that begins after a drug is initiated and resolves after the offending drug is discontinued. This phenomenon was first described in 1945 with sulfadiazine users developing lupus-like symptoms [1]. Since then, multiple forms of DIA have been described, including autoimmune hemolytic anemia, vasculitis, and autoimmune vasculitis, but drug-induced lupus (DIL) remains the most common form of DIA [1]. Of the dozens of agents known to cause DIL, procainamide and hydralazine remain the most common, with an incidence of 20% for procainamide and 5–8% for hydralazine [1, 2].

Hydralazine was first introduced as an antihypertensive in 1951, with the first cases of HIL observed two years later in 1953 [1, 2]. The temporal relationship between initiation of hydralazine and symptoms of HIL varies but tends to be following months to years of treatment. Historically, the incidence of HIL has been dose-related, with a significant increase in the rate of HIL at doses greater than 200 mg daily; however cases have been described at lower doses [2, 3].

## 2. Case Report

**Figure 1:**
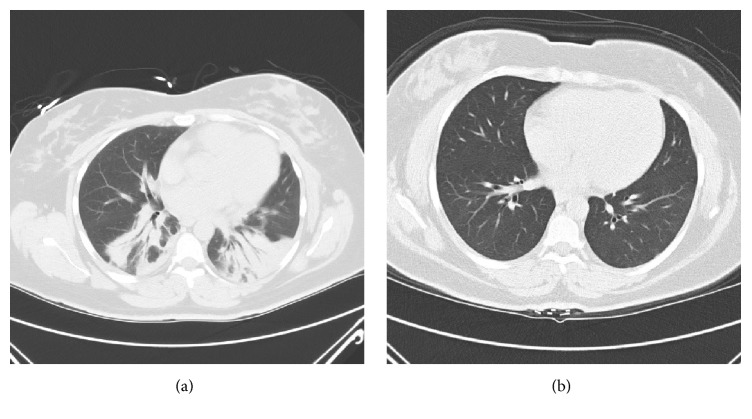
Computed tomography (CT) scans of case subject lungs prior to (a) and after (b) discontinuation of hydralazine therapy.

A 35-year-old African American woman presented to the Emergency Department with progressive shortness of breath. She was found to be hypoxic via home pulse oximeter with an oxygen saturation of 80%. The patient's dyspnea first started two months prior to admission when she was diagnosed and treated for community acquired pneumonia at an outpatient clinic. Her dyspnea progressed despite repeated admissions to community hospitals, during which she had extensive diagnostic tests including pulmonary function tests (PFTs), bronchoscopy, electromyography (EMG), echocardiogram, and computed tomography (CT) scans of the chest. PFTs showed a restrictive pattern with a decreased vital capacity of 21%. Bronchoscopy, EMG, and echocardiogram did not lead to a definitive diagnosis. The chest CT showed nonspecific dense bibasilar consolidation (Figure 1(a)). During each hospitalization, she was managed with antibiotics and steroids for presumed pneumonia with asthma exacerbation. Her most recent admission was one week prior to presentation at which time she was discharged with home oxygen and a CPAP machine.

Past medical history included mild persistent asthma treated with albuterol and budesonide-formoterol, postpartum hypertension treated with hydralazine 10 mg PO every 8 hours and chlorthalidone 25 mg PO daily, environmental allergies treated with cetirizine as needed, gastroesophageal reflux disease treated with esomeprazole, prolactinoma treated with cabergoline, and irritable bowel syndrome. Family and social history were noncontributory.

Review of systems was significant for fevers, chills, productive cough, chest tightness, nausea, abdominal pain, decreased appetite, lower back pain, fatigue, diffuse joint pain, and numbness and tingling of her hands and feet. In the Emergency Department, her oxygen saturation dropped into the 70s on room air with a heart rate of 103 bpm. All other vital signs were stable and within normal limits. On physical examination, the patient appeared to be in moderate distress, gasping for air between words. Crackles were audible in bilateral lower lobes. There was diminished diaphragmatic excursion and abdominal paradox was noted. The remainder of the physical exam was normal. Complete metabolic panel was normal. She had a leukocytosis of 28.6 *∗* 10^9^/L and anemia with hemoglobin and hematocrit of 11.6 g/dL and 35.8%, respectively. Further work-up to support a diagnosis of HIL included ANA, ssDNA, and dsDNA, all of which were negative. ANCA and ACE were also normal. Anti-histone antibodies were elevated at 2.8 units (>1.5 is positive) and her N-acetyltransferase 2 (NAT2) genotype was found to be “intermediate acetylator.”

After a thorough review of the medical history, it was apparent that the onset of symptoms began four weeks after the initiation of hydralazine for hypertension that developed a few weeks postpartum. On day 2 of admission, a presumptive diagnosis of hydralazine-induced lupus (HIL) with pneumonitis was made. Hydralazine was discontinued and pyridoxine 25 mg PO once daily was started for paresthesias. Prednisone 60 mg PO once daily was started due to pulmonary involvement with plans for a prolonged taper. The patient experienced symptomatic improvement within three days of hydralazine discontinuation experiencing improvement in arthralgias and paresthesias and shortness of breath.

After discharge, the patient presented to her outpatient family medicine clinic for follow-up. She reported that her breathing was 80% improved and that she was ambulating without oxygen. She had a repeat chest CT which showed complete resolution of the previous bilateral basilar consolidations (Figure 1(b)).

## 3. Discussion

Hydralazine-induced lupus (HIL) is an adverse effect of hydralazine therapy that has been well-reported in the literature with an incidence estimated at 5–8% per year [1, 2]. Clinical features of HIL include arthralgias, myalgias, pleuritic chest pain, fever, and lymphadenopathy; however, symptoms are nonspecific making certain diagnosis difficult [2]. Compared with systemic lupus erythematosus (SLE), HIL usually has a milder presentation with reversible symptoms after drug discontinuation. While pulmonary manifestations are common in SLE, pulmonary involvement of HIL is rare. Another differentiating feature is the observation that HIL is more likely to have positive anti-histone antibodies (>95% of cases) and negative anti-ds DNA antibodies [2, 3].

Risk factors for HIL include longer duration of therapy (>3 months), total daily dose > 400 mg, female gender, white race, slow acetylation phenotype, and HLA DR4 haplotype [2, 3]. Diagnosis of hydralazine-induced lupus requires establishing a temporal relationship between hydralazine administration and symptom onset, lack of alternative etiologies, and symptom improvement with drug withdrawal [1, 3]. Borchers and colleagues suggested a list of diagnostic criteria to support a diagnosis of drug-induced lupus erythematosus [4]. These criteria include (1) sufficient and continuing exposure to a specific drug, (2) at least one symptom being compatible with lupus, (3) having no history of SLE before using the drug, and (4) resolution of symptoms within weeks to months after discontinuation. These authors note that while a positive ANA is often considered to be required for the diagnosis, a negative ANA does not necessarily eliminate a diagnosis if the patient has other positive autoantibodies.

Our patient is a 35-year-old African American female with past medical history of asthma and postpartum hypertension who presented with shortness of breath and hypoxia after receiving multiple courses of antibiotics and systemic steroids. After determining timing of hydralazine initiation and symptom onset, it was hypothesized that the patient was experiencing hydralazine-induced lupus pneumonitis. While the antibody tests for ANA, anti-dsDNA, and anti-ssDNA were negative, the anti-histone antibody titer was positive and the patient was determined to be an intermediate acetylator. After a course of steroids and discontinuation of hydralazine, the patient's symptoms significantly improved with correlating improvement on CT imaging.

While our patient does not meet the traditional criteria for HIL based on prolonged exposure and high doses, several factors support the diagnosis. The patient had no known history of autoimmune disease prior to initiating hydralazine and did not develop symptoms until several weeks after initiation. Multiple courses of antibiotics and systemic steroids had been completed with worsening of pulmonary functioning, making other potential causes of the pulmonary dysfunction including pneumonia and asthma exacerbation less likely. Pulmonary symptoms improved shortly after hydralazine discontinuation and continued to improve over several weeks. The patient had positive anti-histone antibodies which are known to be associated with drug-induced lupus and the patient was determined to be an intermediate acetylator which is a known risk factor for HIL

When utilizing the Naranjo adverse drug reaction probability scale, this case had a total score of seven making the causality probable. This score was determined based on (1) numerous previous reports of HIL, (2) appearance of the adverse event after hydralazine initiation, (3) improvement in the patient's lung function and arthralgias after drug discontinuation, (4) ruling out alternative causes with several failed treatment courses, and (5) confirmation of the adverse event with CT imaging and positive anti-histone antibodies. As the hydralazine was not titrated or reintroduced after discontinuation, it was not possible to determine if the adverse effect would return upon rechallenge or worsen with dosage increases [5].

Our case is worthy of note due to several features. First, the patient developed lupus-like symptoms relatively quickly after hydralazine initiation with a low total daily dose of 30 mg. While presentation of hydralazine-induced lupus at such a low dose in the acute period has rarely been reported, one case report by Chisholm and colleagues describes a similar presentation. In this case, a 34-year-old African American female started hydralazine 25 mg twice daily as well as hydrochlorothiazide 25 mg twice daily for hypertension. The patient developed severe arthralgias, ocular discomfort, anterior cervical adenopathy, and a fever by day eight of therapy. Hydralazine was discontinued and when symptoms did not improve in two weeks, prednisone was initiated with improvement in symptoms. This case has several similarities to our case as our patient was initiated on a very low dose of hydralazine and developed symptoms acutely (<30 days from medication initiation) [6].

While SLE frequently has pulmonary involvement, drug-induced lupus rarely does with only a few case reports of hydralazine-induced pulmonary infiltrates or pneumonitis [2, 7–10]. In one such case report, Birnbaum and colleagues describe a case of fulminating hydralazine-induced lupus pneumonitis. In their case, a 36-year-old African American female experienced fatigue, dyspnea on exertion, weakness, and arthralgias after receiving hydralazine 100 mg/day for greater than 1 year. Chest CT showed ground glass opacities in bilateral lower lobes and a small pericardial effusion. Hydralazine was discontinued and methylprednisolone was initiated for presumed lupus pneumonitis, but the patient ultimately expired after hypoxia required intubation. Postmortem, the lungs showed diffuse alveolar damage with organization and interstitial infiltrates [10]. Similarities can be drawn with our case due to significant pulmonary involvement with signs of organizing infiltrates that did not respond to antibiotics or steroid courses causing hypoxia.

Our patient also had an antibody profile suggestive of HIL. Preliminary tests for ANA, ANCA, anti-ds DNA, and anti-ss DNA were all negative, which led prior providers to eliminate drug-induced lupus as a potential cause of symptoms. However, anti-histone antibodies were strongly positive which are believed to be more specific for DIL versus SLE. Our patient was also found to be an intermediate acetylator (heterozygous) which is a risk factor for development of hydralazine-induced lupus.

## 4. Conclusions

Our case is an unusual presentation of hydralazine-induced lupus due to the very low total daily dose of hydralazine and the short duration of use prior to symptom onset. Severe pulmonary involvement led to significant morbidity requiring several diagnostic procedures, emergency room visits, hospital admissions, and unsuccessful therapeutic attempts. The value of a complete history is exemplified in this case as the recognition of a temporal relationship with hydralazine initiation and symptom development led to a working diagnosis that was later supported by laboratory evidence. With an accurate diagnosis, appropriate therapy provided improved patient outcomes and reduced healthcare utilization.
